# Procedural Learning through Action Observation: Preliminary Evidence from Virtual Gardening Activity in Intellectual Disability

**DOI:** 10.3390/brainsci11060766

**Published:** 2021-06-09

**Authors:** Alberto Giachero, Agnese Quadrini, Francesca Pisano, Melanie Calati, Cristian Rugiero, Laura Ferrero, Lorenzo Pia, Paola Marangolo

**Affiliations:** 1 Aphasia Experimental Laboratory-Fondazione Carlo Molo Onlus, 10121 Turin, Italy; alberto.giachero@unito.it (A.G.); melanie.calati@gmail.com (M.C.); rugiero.cristian.1980@gmail.com (C.R.); 2 Dipartimento di Psicologia, University of Turin, 10124 Turin, Italy; lorenzo.pia@unito.it; 3 IRCCS Fondazione Santa Lucia, 00179 Rome, Italy; agnese.quadrini@gmail.com; 4 Dipartimento di Studi Umanistici, University Federico II, 80133 Naples, Italy; francescapisano00@virgilio.it; 5 Fondazione Agape dello Spirito Santo Onlus-Villa Lauro, 10132 Turin, Italy; villa.lauro@gruppoagape.it

**Keywords:** intellectual disability, virtual reality, action observation, motor resonance, error learning

## Abstract

Intellectual disability (ID) compromises intellectual and adaptive functioning. People with an ID show difficulty with procedural skills, with loss of autonomy in daily life. From an embodiment perspective, observation of action promotes motor skill learning. Among promising technologies, virtual reality (VR) offers the possibility of engaging the sensorimotor system, thus, improving cognitive functions and adaptive capacities. Indeed, VR can be used as sensorimotor feedback, which enhances procedural learning. In the present study, fourteen subjects with an ID underwent progressive steps training combined with VR aimed at learning gardening procedures. All participants were trained twice a week for fourteen weeks (total 28 sessions). Participants were first recorded while sowing zucchini, then they were asked to observe a virtual video which showed the correct procedure. Next, they were presented with their previous recordings, and they were asked to pay attention and to comment on the errors made. At the end of the treatment, the results showed that all participants were able to correctly garden in a real environment. Interestingly, action observation facilitated, not only procedural skills, but also specific cognitive abilities. This evidence emphasizes, for the first time, that action observation combined with VR improves procedural learning in ID.

## 1. Introduction

Intellectual disability (ID) compromises intellectual and adaptive functioning [1]. It is a clinical condition which appears in the early stages of development, typically in the preschool phase [2].

The etiology can be dependent on multicausal risk factors [3], classified as genetic or acquired causes [4,5]. Genetic factors include chromosomal or hereditary disorders [4,6], whereas non-genetic causes include prenatal (chromosomal disease, congenital errors of metabolism, brain malformations, and maternal disease), perinatal (events related to labor or delivery leading to neonatal encephalopathy), and postnatal factors (hypoxia, infection, brain injuries, convulsive disorders, toxic-metabolic intoxication) [3,7,8].

However, overcoming this extreme variability, all these factors increase the risk of neurological disorder [9,10]. Thus, they contribute to damage to the central nervous system, interfering with ongoing developmental cascades [11].

Intellectual disability can impair a person’s cognitive profile with different degrees of severity, ranging from mild through moderate to severe [12].

People with ID manifest a cognitive delay in the expected stages of development, with difficulty in logical reasoning [13]. Executive functions such as problem solving, planning, abstract thinking, judgment, and learning from experience are often severely compromised [1]. Accordingly, these persons manifest a loss of independence and autonomy in one or more aspects of daily life [14,15]. Indeed, together with executive function difficulties, problems in the motor, language, and social domains are often present, which also affect adaptive functionality [10]. According to the American Association on Intellectual and Developmental Disabilities [1], deficits in adaptive functioning refer to how well a person meets cultural and age-related standards of independence and social responsibility. Adaptive functioning involves adaptive reasoning in the following domains: practical, conceptual, and social. The conceptual domain involves language, writing, reading, math reasoning, memory, acquisition of practical knowledge, problem solving, and judgment in novel situations [16,17,18,19,20,21,22,23]. The social domain refers to empathy; awareness of others’ thoughts, feelings, and experiences; communication skills; social judgment; and the ability to make and maintain friendships. The practical domain involves learning and self-management across life settings, including job responsibilities; personal care; managing money; leisure activities, the ability to manage one’s behavior; and task organization [13,24,25,26,27]. Learning and knowledge of these subjects seem not to be available to the conscious awareness, although learned skills can be processed automatically and rapidly. Indeed, the process of learning acquisition is relatively slow, with a fair amount of repetition or practice required for the ID subject to automate the process [1].

To date, the great heterogeneity in etiology, severity, and cognitive profile in ID has made it difficult to establish universal principles for planning effective treatment approaches [28]. Classical occupational therapy has been proposed to foster ID independence, to improve functional activity in daily life through methodical and repetitive training of specific tasks [29]. Repetitive practice embraces the use of prompts [29], classified as general instructions or cues [28]. Generally, prompting methods are implemented with the usage of time delay (TD) or constant time delay (CTD) supporting the acquisition of skills or sequential learning [30,31]. Several studies have also focused on the rehabilitation of motor disorders [32,33,34,35]. Indeed, sensory integrative (SI) therapy and/or the perceptual-motor (PM) approach are largely based on the idea that an improvement of the motor and sensory skills will also exert its influence in the cognitive domain, thus, improving cognitive functioning in ID [36]. Accordingly, the emerging viewpoint of embodied cognition is that the motor, sensory, and cognitive systems closely interact and cognitive processes are deeply rooted in the body’s interactions with the world [37]. Hence, the body has a fundamental role in human cognition that is expressed through its sensorimotor pathways [37,38,39,40], which are already active at 9 months old [41]. Within this approach, it is now a well-accepted notion that the observation of actions performed by others activates in the perceiver the same sensorimotor structures responsible for the actual execution of those same actions [42]. Thus, while observing other people performing everyday actions, neural structures involved in the actual execution of those actions are recruited in the observer’s brain as if he/she were actually performing the observed action [43]. This motor resonance relates to understanding, imitation learning, and predicting action outcomes [42,43,44,45,46]. To date, several studies have consistently shown that action observation (AO) is an effective way to learn or enhance the performance, particularly in the motor [43,47,48] and language domain for the recovery of aphasia [49,50,51]. AO therapy has also been applied to ID subjects with genetic etiology [52,53,54], outlining specific profiles of learning in the different selected syndromes [53]. Indeed, the authors of [53] have shown an opposite pattern of visuo-motor sequence learning in Williams (WS) and Down (DS) syndrome. Subjects with WS benefited from observational training, while subjects with DS were severely compromised in learning by observation.

In these last years, the development of new technologies has maximized the efficacy of these therapeutic approaches [28] using smartphones, computer programs, and tablets, which can potentiate the autonomous acquisition of a task at the individual’s own pace [55,56].

Although the use of virtual reality (VR) has long been considered as a useful tool [57], primarily in the development of videogames, only in the past decade has it re-emerged as a promising adjuvant treatment strategy for cognitive rehabilitation [58,59,60]. VR implies the use of a computer-generated simulation of 3D environments, through which the users can experience a full or semi-immersive experience, as if they were in a real context dimension [58,61,62]. Innovative VR devices, including head mounted displays (HMDs) [57,63,64,65], give the opportunity of taking advantage of a higher level of immersion (i.e., immersive virtual environments (IVEs)) [57], which is effective for enhancing motor and cognitive skills in healthy [63,65,66] and clinical populations [67,68,69]. Conversely, semi-immersive VR, due to the lack of HDMs, does not guarantee a full immersion but favors the interaction among participants, promoting group therapies [61]. Although different studies have suggested that neurological subjects, even in the acute stage, are successful stimulated by outdoor interventions [70], in rehabilitation programs for ID, VR can provide a safe setting through which the users can practice skills which would be dangerous in the real world [71,72]. Indeed, VR scenarios can be manipulated in ways which are not always feasible for disabled people in everyday life [71,72].

Usually, an individual entering a virtual environment feels a part of this world, and she/he can interact with it almost as she/he would do in the real world [73]. The engagement of the sensorimotor skills allows, therefore, implementing specific cognitive, behavioral, and adaptive functions [58]. Regarding movement limitations, it has been shown that virtual reality offers the possibility of developing new sensorimotor strategies, useful for motor recovery [74]. The interaction in three dimensional scenarios and the simultaneous observation of avatar movements seem to activate the AO system and induce brain reorganization [46,75]. It has also been suggested that observing the errors made in the actions of an avatar activates the onlooker’s error detection brain system [76], which has a fundamental role in skill learning [77]. Indeed, Nishizawa et al. [78] have shown that the acquisition of correct movements can be achieved by comparing the observation of a model with self-observation. Indeed, the mismatch between these two conditions seems to act as a feedback that helps the brain to produce an adjustment [77]. According to Standen et al. [71], virtual environments may help subjects with ID to acquire the necessary skills for independent living by improving cognition, practice, and social skills. Accordingly, Brookes and colleagues [79] have investigated the role of VR training for learning food preparation in a group of ID subjects. Results showed that the VR context was more advantageous for the acquisition of real task performance compared to workbook training [79].

Here, we report a video-based action observation treatment which makes use of a semi-immersive VR environment, to investigate its therapeutic benefits in enhancing gardening skills in a group of fourteen ID participants.

## 2. Materials and Methods

### 2.1. Participants

A total of fourteen participants (ten females and four males) with intellectual disability were recruited from the Villa Lauro Foundation in Turin, where they lived in a residential or semi-residential regime at the time of the study. They were aged between 26 and 67 years (47.7+/−14.08), with an educational level of 5 to 13 years (8.07+/−2.43), and presented with cognitive, intellectual, and adaptive functioning impairments. The inclusion criteria were Italian language as mother tongue, right-handed (Edinburgh test [80]), perinatal etiology, and no hearing loss (screened via pure tone audiometry). Subjects with a diagnosis of Down, Fragile x, Rett syndrome, or autism were excluded. According to the Wechsler adult intelligence scale (WAIS-R test, [81]), the fourteen participants were assigned to three different groups, classified as mild with an average IQ score of 66.25 (range 63 to 68; DS: +/−2.75; WAIS-R range: 60–69), moderate with an average IQ score of 55.4 (range 52 to 58; DS: +/−2.7; WAIS-R range: 59–45), and severe with an IQ score < 45 (see Table 1).

All subjects presented with difficulties in different everyday life activities, particularly with learning gardening skills.

### 2.2. Ethical Approval

The data analyzed in the current study are conformed with the Helsinki Declaration. Our named institutional review board (Ethical Committee, University of Turin) specifically approved this study (protocol 256112) with the understanding and written consent of each subject.

### 2.3. Materials and Apparatus

**Figure 1:**
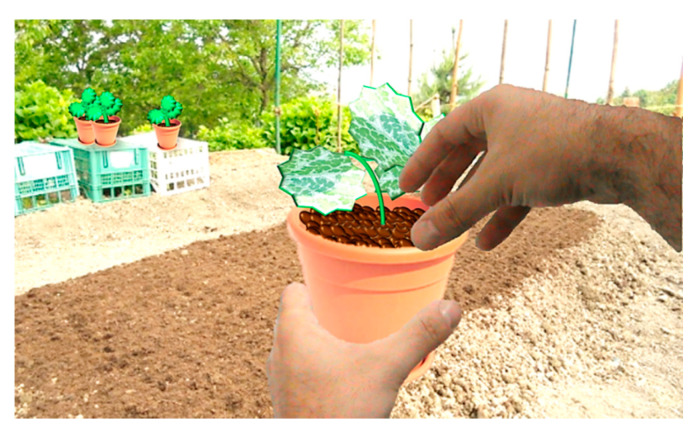
Example of a VR scenario showing how to take out the plant from the pot.

The semi-immersive VR scenarios were projected on a screen (50 inches). They were created with a NeuroVR 2.0 open-source software (http://www.neurovr2.org) by the authors of the present study from the Aphasia Experimental Laboratory of Carlo Molo Onlus Foundation in Turin. To favor the interaction between patients within the therapeutic setting, the authors opted for a semi-immersive virtual reality condition in which no patient wore a helmet. To limit the window effect typical of a non-immersive virtual reality condition, a 50-inch curved screen was used to guarantee a sufficient level of image depth and sense of immersion for each patient [82]. The apparatus projected different virtual scenarios related to the different steps required to learn the sowing of zucchini (Figure 1). During the video projection, different cognitive exercises were proposed to the subject, aimed at supporting the interaction of the participant with the VR environment, such as semantic association tasks (i.e., association between the pictures of the tools required for sowing zucchini and their names (i.e., spot, soil, zucchini seeds), phonological fluency tasks (i.e., subjects were required to produce words beginning with the last letter of the previous word: ‘pencil→lemon’), and attentional tasks (i.e., subjects had to compare two matrices of objects placed on different spatial positions and select the missing object).

### 2.4. Clinical Data

The assessment of each subject’s profile investigated three different domains: intelligence, cognitive abilities, and practical functioning. As previously reported, intelligence was assessed using the WAIS-R scale [81]. The mini mental state examination (MMSE) (cut-off score < 24; [83]) was administered to evaluate the participant’s global cognitive functioning. In addition, subjects were presented with specific tests aimed at investigating different cognitive domains: the frontal assessment battery (FAB; cut-off score < 13.50; [84]), the semantic (cut-off score < 7.25), and phonemic fluency test (cut-off score < 7.25; [85]) for evaluating executive functions abilities; the token test for investigating language comprehension (cut-off score < 26.50; [85]); and attentive matrices were administered (cut-off score < 31; [85]) to evaluate selective attention. Short-and-long term verbal memory were measured through the verbal span test (cut-off score < 3) and the prose memory test (cut-off score < 4.75), while Corsi’s test (cut-off scores < 3.75) and the supra-span spatial task (cut-off scores < 5.75) [85] were administered for testing short-and-long term spatial memory. In addition, the participant’s ability to sow zucchini was assessed through a questionnaire, which was completed by three caregivers from the foundation who take care of the subjects, before starting the VR training. The questionnaire consisted of twelve items which investigated the participants’ acquired knowledge of the different steps required for sowing the zucchini. For example, ‘the participant spontaneously selects the tools necessary for zucchini sowing’, ‘the participant spontaneously takes the pot for planting’, and ‘the participant is not able to recognize the pot as a useful tool for sowing, even after suggestion’ were among the items to be scored. Each item was measured on five-point levels of a Likert scale: strongly disagree (1), disagree (2), neither agree nor disagree (3), agree (4), and strongly agree (5). Total score ranged from 12 to 60, with higher scores representing the correct execution of each step.

### 2.5. Procedure

Before starting the VR training, each subject was placed in front of a table where there were both the tools for sowing zucchini (e.g., pot, soil, zucchini seeds, watering can, fertilizer, hoe, rake, gloves), and some distractors (a pinecone, sticks, knife, hammer, beans, spoon, other seeds). An operator asked the participants to sow the zucchini and walked away leaving the participant alone. While the participant was doing the task, he/she was recorded with a video camera. Once these recordings were completed, the subjects were divided into three groups based on their IQ score from the WAIS-R test. Each group underwent fourteen weeks of training with two training sessions per week, each lasting one hour. In the first session, participants were asked to carefully observe the VR video where the correct procedure of the different stages for sowing zucchini was projected and to perform the different cognitive exercises. For example, the first phase of sowing consisted of putting the soil into the pot with a shovel, and each participant had to point to the correct tool presented among five alternatives (i.e., rake, spoon, pinecone, stick, knife). During each session, the therapist encouraged the group members to interact with each other and to take turns commenting on what they were observing. In the second weekly session, the subjects looked at their previous recordings, in which the different stages of sowing were performed incorrectly. This phase had the purpose of allowing the subjects to grasp the discrepancy between their incorrect performance and the correct procedure projected in the VR video. At the end of the fourteen weeks, each participant was again placed in front of the table and asked to sow the zucchini without any assistance from the operators and without observing the virtual video.

**Figure 2:**
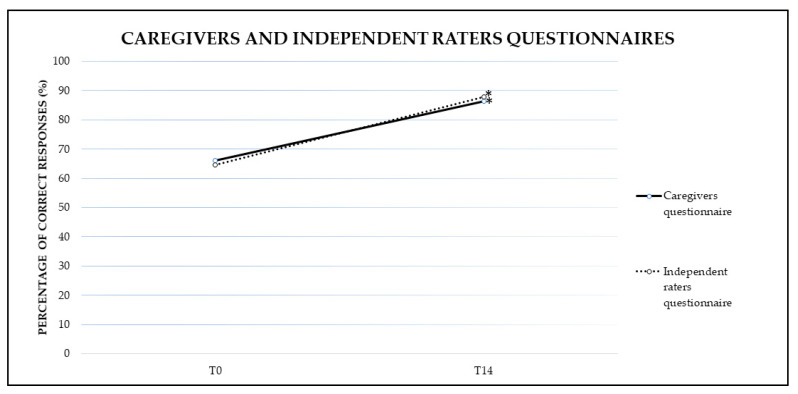
Mean percentage of correct responses in the questionnaire administered by the caregivers and independent raters at baseline (T0) and at the end of the treatment (T14). Sig. mixed ANOVA design: * *p* ≤ 0.05.

The tests and the questionnaire were given to the subjects and the caregivers, respectively, at the beginning and at the end of the VR training (see Figure 2 for results). To minimize subjective judgements, the questionnaire was also given to three independent raters.

### 2.6. Data Analysis

All statistical analyses were conducted with IBM SPSS Statistics 22 software.

For each neuropsychological test and for the questionnaire administered to the caregivers and the independent raters, a two-way mixed analysis of variance (ANOVA) with one between-subjects factor (GROUP (1-Mild vs. 2-Moderate vs. 3-Severe)) and one within-subjects factor (TIME (baseline (T0) vs. last day (T14)) was performed on the mean percentage of correct responses at two time points (T0 vs. T14). To directly compare the mean percentage of correct responses given by the caregivers and the independent raters, before and after the treatment, two paired *t*-tests were performed (T0 vs. T14).

A post hoc Bonferroni test was conducted on the significant effects observed in the ANOVA. *p*-values < 0.05 were considered as statistically significant.

To verify the applicability of the above parametric analysis, a Shapiro–Wilk normality test was applied, which revealed a normal distribution of the data.

## 3. Results

### 3.1. Caregivers and Independent Raters Questionnaires

#### 3.1.1. Caregivers Questionnaire

The analysis showed a significant effect of TIME (F(1,11) = 12.01, *p* = 0.01, *η*^2^ = 0.55). No significant differences emerged for GROUP (F(2,11) = 0.67, *p* = 0.54, *η*^2^ = 0.12). The interaction GROUP × TIME was not significant (F(2,11) = 0.30, *p* = 0.80, *η*^2^ = 0.05). Indeed, for TIME a significantly greater percentage of correct responses at T14 (86.40%) were found compared to T0 (66.15%) (Figure 2).

#### 3.1.2. Independent Raters Questionnaire

The analysis showed a significant effect of TIME (F(1,11) = 13.33, *p* = 0.00, *η*^2^ = 0.60). No significant differences emerged for GROUP (F(2,11) = 1.32, *p* = 0.31, *η*^2^ = 0.21). The interaction GROUP × TIME was not significant (F(2,11) = 0.20, *p* = 1.00, *η*^2^ = 0.04). Indeed, for TIME a significantly greater percentage of correct responses at T14 (87.82%) were found compared to T0 (64.49%) (Figure 2).

No significant differences emerged in the mean percentage of correct responses given by the caregivers and the independent raters before (T0(1,13) = 0.443, *p* = 0.666) and after the treatment (T14(1,13) = −0.413, *p* = 0.687).

### 3.2. Neuropsychological Tests

#### 3.2.1. Phonemic Fluency

**Figure 3:**
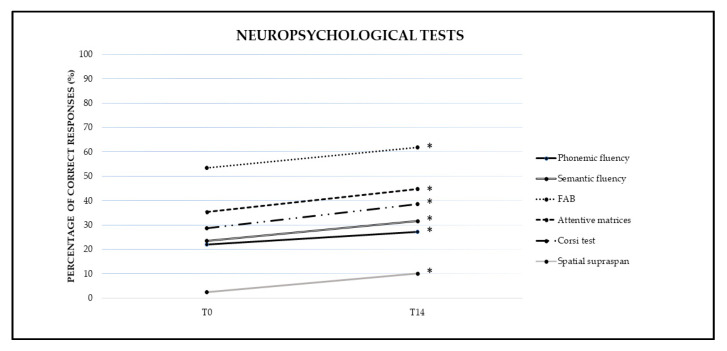
Mean percentage of correct responses for each neuropsychological test at baseline (T0) and at the end of the treatment (T14). Sig. mixed ANOVA design: * *p* ≤ 0.05.

The analysis showed a significant effect of TIME (F(1,11) = 5.73, *p* = 0.04, *η*^2^ = 0.34) and of GROUP (F(2,11) = 9.78, *p* = 0.00, *η*^2^ = 0.64). The interaction GROUP × TIME was not significant (F(2,11) = 1.03, *p* = 0.39, *η*^2^ = 0.16). Indeed, for TIME a significantly greater percentage of correct responses at T14 (27.09%) were found compared to T0 (22.00%). Bonferroni’s post hoc test revealed that the Mild group performed significantly better than the Moderate (Group 1 vs. Group 2, *p* = 0.04) and the Severe groups (Group 1 vs. Group 3, *p* = 0.00) (see Figure 3).

#### 3.2.2. Semantic Fluency

The analysis showed a significant effect of TIME (F(1,11) = 9.71, *p* = 0.01, *η*^2^ = 0.47) and GROUP (F(2,11) = 9.41, *p* = 0.00, *η*^2^ = 0.63). The interaction GROUP × TIME was not significant (F(2,11) = 2.95, *p* = 0.09, *η*^2^ = 0.35). Indeed, for TIME a significantly greater percentage of correct responses at T14 (31.72%) were found compared to T0 (23.45%). Bonferroni’s post hoc test revealed that the Mild group performed significantly better than the Severe group (Group 1 vs. Group 3, *p* = 0.00) (see Figure 3).

#### 3.2.3. Frontal Assessment Battery (FAB)

The analysis showed a significant effect of TIME (F(1,11) = 10.05, *p* = 0.01, *η*^2^ = 0.50) and GROUP (F(2,11) = 5.60, *p* = 0.02, *η*^2^ = 0.50). The interaction GROUP × TIME was not significant (F(2,11) = 1.10, *p* = 0.40, *η*^2^ = 0.20). Indeed, for TIME a significantly greater percentage of correct responses at T14 (61.75%) were found compared to T0 (53.31%). Bonferroni’s post hoc test revealed that the Mild group performed significantly better than the Severe group (Group 1 vs. Group 3, *p* = 0.02) (see Figure 3).

#### 3.2.4. Attentive Matrices

The analysis showed a significant effect of TIME (F(1,11) = 15.94, *p* = 0.00, *η*^2^ = 0.61) but no significant differences emerged for GROUP (F(2,11) = 1.9, *p* = 0.20, *η*^2^ = 0.27). The interaction GROUP × TIME was not significant (F(2,11) = 0.77, *p* = 0.50, *η*^2^ = 0.13). Indeed, for TIME a significantly greater percentage of correct responses at T14 (44.68%) were found compared to T0 (35.39%) (see Figure 3).

#### 3.2.5. Corsi Test

The analysis showed a significant effect of TIME (F(1,11) = 9.51, *p* = 0.01, *η*^2^ = 0.51) but no significant differences emerged for GROUP (F(2,11) = 3.8, *p* = 0.06, *η*^2^ = 0.45). The interaction GROUP × TIME was not significant (F(2,11) = 0.46, *p* = 0.64, *η*^2^ = 0.09). Indeed, for TIME a significantly greater percentage of correct responses at T14 (38.58%) were found compared to T0 (28.66%) (see Figure 3).

#### 3.2.6. Spatial Supra-Span

The analysis showed a significant effect of TIME (F(1,11) = 4.90, *p* = 0.05, *η*^2^ = 0.32) but no significant differences emerged for GROUP (F(2,11) = 1.47, *p* = 0.27, *η*^2^ = 0.22). The interaction GROUP × TIME was not significant (F(2,11) = 1.11, *p* = 0.36, *η*^2^ = 0.20). Indeed, for TIME a significantly greater percentage of correct responses at T14 (10.12%) were found compared to T0 (2.43%) (see Figure 3).

#### 3.2.7. Token Test

The analysis did not show a significant effect of TIME (F(1,11) = 3.27, *p* = 0.10, *η*^2^ = 0.25) and of GROUP (F(2,11) = 3.01, *p* = 0.10, *η*^2^ = 0.38). The interaction GROUP × TIME was not significant (F(2,11) = 0.39, *p* = 0.69, *η*^2^ = 0.07).

#### 3.2.8. Verbal Span

The analysis did not show a significant effect of TIME (F(1,11) = 1.81, *p* = 0.20, *η*^2^ = 0.14) but significant differences emerged for GROUP (F(2,11) = 7.60, *p* = 0.01, *η*^2^ = 0.60). The interaction GROUP × TIME was not significant (F(2,11) = 1.03, *p* = 0.39, *η*^2^ = 0.16). Bonferroni’s post hoc test revealed a significant difference between the Mild and the Severe group (Group 1 vs. Group 3, *p* = 0.01).

#### 3.2.9. Prose Memory Test

The analysis did not show a significant effect of TIME (F(1,11) = 3.44, *p* = 0.10, *η*^2^ = 0.23). Significant differences emerged for GROUP (F(2,11) = 5.40, *p* = 0.02, *η*^2^ = 0.50). The interaction GROUP × TIME was not significant (F(2,11) = 0.55, *p* = 0.60, *η*^2^ = 0.01). Bonferroni’s post hoc test revealed that the Mild group performed significantly better than the Severe group (Group 1 vs. Group 3, *p* = 0.02).

#### 3.2.10. Mini Mental State Examination (MMSE)

The analysis showed a significant effect of TIME (F(1,11) = 6.60, *p* = 0.03, *η*^2^ = 0.40) and of GROUP (F(2,11) = 4.00, *p* = 0.05, *η*^2^ = 0.42). The interaction GROUP × TIME was not significant (F(2,11) = 1.00, *p* = 0.43, *η*^2^ = 0.14). Indeed, for TIME a significantly greater percentage of correct responses at T14 (67.17%) were found compared to T0 (58.83%). Bonferroni’s post hoc test revealed that the Mild group performed significantly better than the Severe group (Group 1 vs. Group 3, *p* = 0.05).

## 4. Discussion

The present study investigated the efficacy of a video-based action observation treatment projected using a semi-immersive VR environment for enhancing the gardening skills in fourteen people with different severities of ID. Our results clearly showed the positive impact of this treatment. Indeed, both the caregivers and the independent raters agreed on the acquisition of zucchini sowing after fourteen days of action observation treatment in all participants. Thus, this finding suggests that the VR training may have positively affected the skill learning, regardless of the degree of intellectual disability. These results fit well with the well-known theory that considers observational learning as a main strategy through which children develop their motor and cognitive abilities [86]. Indeed, children spontaneously imitate different actions, speech patterns, and the use of objects to complete tasks. Imitative learning is valuable because the behavioral action of others ‘like me’ serves as a proxy for the self [87]. Thus, observation accelerates the observer’s acquisition of complex tasks and actions, limiting the time-consuming process of learning by doing [88,89].

Previous studies have investigated the impact of action observation (AO) on learning procedural skills in ID subjects with genetic etiology [52,53,54]. Interestingly, the authors found that different genetic profile, brain morphology, and functionality entailed specific profiles of learning in the different ID syndromes. In contrast to subjects with Down syndrome, who benefited from learning by doing, individuals with Williams syndrome benefited from observational training, while they were severely impaired in learning by doing [53].

In our study, although some differences emerged in the cognitive tests, with the less severe ID group (Mild group) showing, in general, a better performance compared to the other two groups (which, due to the cognitive load required by the cognitive tasks, was partly expected), all subjects reached the same level of ability in sowing.

The role of AO as an effective strategy for motor and cognitive enhancement has also been supported more recently by several studies, which showed that AO might promote motor [43,47,48] and language recovery [49,50,51] in neurological populations. Accordingly, the embodied cognition view holds that cognitive processes are deeply rooted in the body’s interaction with the world. Hence, human cognition, rather than being centralized and sharply distinct from peripheral input and output modules, is closely related to sensorimotor processing. Within this approach, a related consolidated finding was also that the observation of actions performed by another person activates the same sensorimotor structures, as if the perceiver were executing those actions [40,41,42,43,90].

Thus, the activation of sensorimotor processes through AO can, in turn, enhance motor and language learning [43,49,50]. Although we did not collect neuroimaging data in our study, it is reasonable to assume that during the observation of the sowing procedures, the participants’ sensorimotor system was activated as they were performing the same procedures and that this in turn facilitated the acquisition of the gardening skills. Given that our cognitive tests were only aimed at selecting the correct tools required for sowing and not their usage, we believe that AO had a specific effect on learning the sowing procedures. Moreover, since our participants were all affected by congenital syndromes, this allows us to exclude that the results obtained were simply due to spontaneous recovery. Indeed, according to the neuroconstructivist view, atypical development should not be considered as a catalogue of impaired and intact functions, in which only impaired modules develop atypically, but as a clinical condition affecting the entire cognitive functioning [91].

Together with observational learning, several authors have also stressed the crucial role played by error detection processes in skill learning [71,76,77,78]. Nishizawa and collaborators [78] have shown that the acquisition of correct movements can be achieved by comparing the observation of a model with self-observation. Indeed, the mismatch between these two conditions seems to act as a feedback that helps the brain to produce an adjustment [77]. Thus, in our study, the possibility for the participants to compare the VR-scenarios with the recordings of their own errors while sowing further helped the learning process and enhanced the participants’ performance.

As stated in the Introduction, different studies have shown that people with ID have problems in lexical access, working memory, and executive control, including shifting between tasks [92,93,94,95]. Relevantly, the neuropsychological tests administered after the training showed a significant improvement in all participants in the different cognitive domains. After the treatment a greater performance was present in executive functions tasks (phonemic and semantic fluency; FAB test) in all groups, although the mild group performed, in general, better than the other two groups. Since these tests involve complex executive and verbal abilities, these differences were expected [92]. Interestingly, the three ID groups also showed the same amount of improvement in attention and short- and long-term spatial memory tasks. Thus, the VR videos trained not only the participants’ gardening skills but had a significant impact on tasks such as executive functions and attentional and spatial skills that were closely related to the observed procedures.

Finally, at the end of the treatment, we did not observe significant changes in the cognitive tests that measured specific verbal abilities such as verbal comprehension of complex commands (token test) and verbal short-and long-term memory. Since the memory load required by these tests is high and these skills were not specifically trained during the VR-treatment, these results were expected. Indeed, our VR training was aimed at improving gardening abilities, thus, it was reasonable to find an improvement only in the tests related to the practiced skills (i.e., executive functioning and spatial memory).

## 5. Conclusions

In conclusion, although our results are encouraging for identifying treatment protocols for ID subjects, we are aware that they have some limitations due to the small sample size considered and the absence of a control group. Indeed, we know that to reach definitive conclusions for our action observation training, we need to replicate the same effects in a largest group. It would also be interesting to compare these findings with those obtained with training to better understand the real effectiveness of our treatment. However, apart from these limitations, we believe that research concerning ID is crucial to promote the acquisition of procedural skills that would allow improving functional abilities and cognitive recovery in this population.

## Figures and Tables

**Table 1:** Demographic and clinical data of the fourteen participants.

Group	Participants	WAIS-R	Sex	Age	Educational Level	Etiology
1 Mild	1	69	F	72	8	Neonatal cerebropathy
1-Mild	2	63	F	56	8	Neonatal cerebropathy
1-Mild	3	65	F	68	5	Neonatal cerebropathy
1-Mild	4	68	F	52	8	Perinatal cerebropathy
2-Moderate	5	57	M	36	8	Neonatal cerebropathy
2-Moderate	6	57	M	44	8	Perinatal cerebropathy
2-Moderate	7	53	F	59	5	Epileptogenic encephalopathy
2-Moderate	8	52	F	28	8	Neonatal cerebropathy
2-Moderate	9	58	F	61	5	Perinatal asphyxia
3-Severe	10	<45	M	38	13	Perinatal asphyxia
3-Severe	11	<45	F	34	8	Neonatal cerebropathy
3-Severe	12	<45	F	31	13	Neonatal cerebropathy
3-Severe	13	<45	M	42	8	Perinatal cerebropathy
3-Severe	14	<45	F	47	8	Perinatal asphyxia

## Data Availability

The data presented in this study are contained within the article.
